# Enhancing physicochemical, bioactive, and nutritional properties of sweet potatoes: Ultrasonic contact drying with slot jet nozzles compared to hot-air drying and freeze drying

**DOI:** 10.1016/j.ultsonch.2024.107216

**Published:** 2024-12-30

**Authors:** Gulcin Yildiz, Yuan Gao, Junzhou Ding, Si Zhu, Guibing Chen, Hao Feng

**Affiliations:** a Department of Family and Consumer Sciences, North Carolina A&T State University, Greensboro, NC 27411, USA; b Department of Food Engineering, Igdir University, Iğdır 76000, Turkey; c Center for Excellence in Post-Harvest Technologies, North Carolina A&T State University, The North Carolina Research Campus, 500 Laureate Way, Kannapolis, NC 28081, USA

**Keywords:** Ultrasonic-contact drying, Sweet potato, Nutrition, Rehydration, Color, Heatmap

## Abstract

Sweet potatoes are a rich source of nutrients and bioactive compounds, but their quality can be impacted by the drying process. This study investigates the impact of slot jet reattachment (SJR) nozzle and ultrasound (US) combined drying (SJR + US) on sweet potato quality, compared to freeze-drying (FD), SJR drying, and hot air drying (HAD). SJR + US drying at 50 °C closely resembled FD in enhancing quality attributes and outperformed HAD and SJR in key areas such as rehydration, shrinkage ratios, and nutritional composition. Notably, SJR + US at 50 °C produced the highest total starch (36.84 g/100 g), total dietary fiber (8.48 g/100 g), total phenolic content (158.19 mg GAE/100 g), total flavonoid content (119.08 mg QE/g), DPPH antioxidant activity (6.44 μmol TE/g), β-carotene (31.98 mg/100 g), and vitamin C (5.27 mg/100 g). It also exhibited higher glass transition temperatures (Tg: 14.49 °C), indicating better stability at room temperature. The hardness values for SJR + US samples were similar to FD, while HAD samples had the highest hardness. SJR + US at 50 °C resulted in the lowest total color changes (ΔE), indicating minimal impact on appearance. Additionally, FTIR analysis revealed that peaks in specific spectral regions indicated superior preservation of bioactive compounds in SJR + US samples compared to other methods, which was also confirmed by principal component analysis (PCA) and heatmap visualization. Overall, these findings suggest that SJR + US is an effective alternative to conventional drying techniques, significantly improving the quality of dried sweet potatoes.

## Graphical abstract

**Figure:**
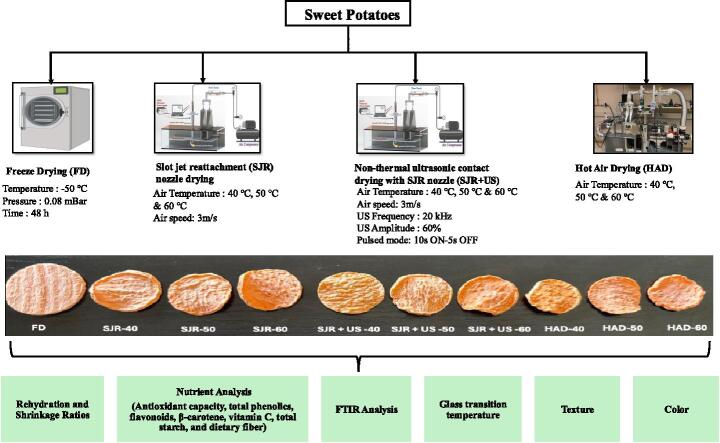

## 1 Introduction

Sweet potatoes (*Ipomoea batatas* L.) are important root vegetables abundant in fiber, starch, vitamins, and functional biological compounds. They are recognized for their carotenoids, phytochemicals, and beneficial properties including anticancer and antimicrobial effects, which contribute positively to human health [9]. Sweet potatoes can be consumed raw or processed into staple foods, snacks, or bakery products. However, because of their abundance of moisture, they are prone to microbial activity, leading to degradation and spoilage. Additionally, since sweet potatoes are seasonal, they cannot maintain optimal quality for extended post-harvest periods. Therefore, they are often used shortly after harvesting or preserved through hot air drying (HAD) [30].

HAD is commonly employed in industrial and commercial settings for preservation of food and agricultural products. This technique utilizes circulating hot air within a chamber to remove moisture, facilitated by a fan or blower and an electric or gas heater [41]. However, HAD is known to consume substantial amounts of energy and time and can negatively impact the overall quality of the finished product [13]. Over the years, several hybrid drying methods have emerged as feasible alternatives to HAD for drying agricultural products, such as combining microwave and infrared (IR) energy with HAD [42], [63]. Despite being innovative, improper application of these thermal techniques can also lead to degradation in product quality. Hence, there is an ongoing necessity to innovate and develop novel drying methods or alternative energy sources for food product drying.

Impinging jet nozzles, known for their ability to provide intense localized heat and mass transfer, have found applications in baking and drying operations. The slot jet reattachment (SJR) nozzle is a specialized type of jet that can regulate the impingement force exerted on a surface by the jet flow. In this design, the jet exits the nozzle and reattaches to a nearby adjacent surface. At the edges of the free stream, turbulent mixing triggers secondary circulation through entraining mass, resulting in the flow reattaching to the surface in an oval shape near the nozzle [25]. On the other hand, ultrasonic contact drying is a non-thermal drying technique that utilizes high-frequency vibrations to remove water from moist materials, including food products [29]. During the drying process, when moisture content is high, high-frequency sound waves (20–100 kHz) generated by piezoelectric transducers can create a mist on the surface of the product. A stream of air then carries away the mist and vapor, effectively drying the material [35]. Ultrasonic contact drying has been investigated for the drying of fruits, plant proteins and distillers dried grains with solubles (DDGS) [28], [35], [29]. Results have shown that ultrasonic contact drying can significantly reduce drying time and improve product quality compared to conventional HAD, especially for heat-sensitive materials. Combining the SJR nozzle with ultrasonic contact drying can enhance drying efficiency by coupling the intense localized heat and mass transfer on produce surfaces from the SJR nozzle with the rapid moisture removal capabilities of ultrasonic vibrations. This synergy can lead to faster drying rates, reduced energy consumption, and improved product quality, which is explored in this study.

Limited studies have used ultrasound as a pre-treatment for sweet potatoes, followed by freeze drying or HAD, to achieve low-moisture final products [48], [62]. No study has yet explored the combination of slot jet reattachment (SJR) with ultrasonic contact drying for sweet potatoes. This study evaluates the effectiveness of combining SJR and ultrasound at different temperatures (40, 50, and 60 °C) and compares it with freeze drying (FD) by focusing on the physiochemical, bioactive and nutritional characteristics of dried sweet potatoes, including rehydration and shrinkage ratios, color, glass transition temperature, texture, antioxidant capacity, total phenolics, flavonoids, β-carotene, vitamin C, total starch, and dietary fiber. Principal component analysis (PCA) and heatmap analysis were performed to assess the nutritional properties and visualize the distribution of components across treatments.

## 2 Material and methods

### 2.1 Sample preparation

Fresh sweet potatoes from a Food Lion in Kannapolis, NC, were stored at 4 ± 0.5 °C until use. Initial moisture content (81.8 %, w.b.) was determined following method 930.15Association of Official Analytical Chemists [2] International using oven drying (DVS 402, Yamato, Tokyo, Japan) at 105 ± 5 °C. Samples were sliced to 3.0 mm thickness and 37.0 mm diameter for further processing. The drying experiments were performed until the final moisture content of the samples reached approximately ≤6 %.

### 2.2 Drying experiments

The sweet potatoes were dried with SJR alone, non-thermal ultrasonic contact drying + SJR nozzle (SJR + US), and hot air drying (HAD) alone. The effectiveness of these methods was compared to that of freeze drying (FD).

**Fig. 1:**
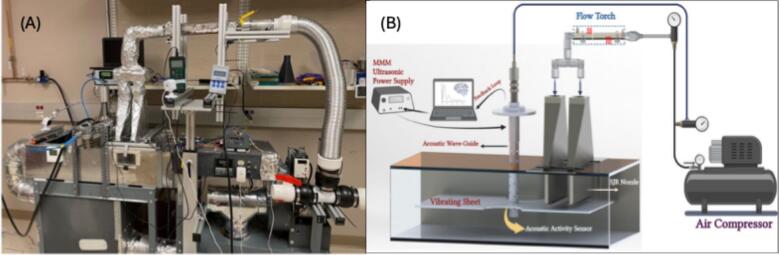
Ultrasonic contact drying with SJR nozzle. (A) Photo of the drying prototype, including SJR nozzle and hot air circulation chamber and (B) schematic showing the arrangement of MMM ultrasound drying surface and SJR nozzle.

*SJR and SJR + US drying:* The SJR + US and SJR drying of sweet potatoes were performed with a non-thermal ultrasonic contact drying and SJR combined drying prototype, as illustrated in Fig. 1. This system includes an ultrasonic drying plate housed in a drying chamber, a multi-frequency, multimode, modulated (MMM) ultrasonic generator, an air circulation pump, a heater with a temperature controller, and a computer-aided controller for regulating ultrasound frequency and power, in addition to the SJR nozzle. Sweet potato samples, with a diameter of 37 mm and a uniform thickness of 3 mm, were placed on the ultrasonic vibrating plate. The ultrasonic power was set at 60 % of its maximum amplitude, and the generator operated the vibrating plate at 20 kHz. The ultrasound operated in a pulsed mode (10 s on – 5 s off) to prevent overheating of the piezoelectric transducer and reduce energy consumption. For SJR drying, the same drying device was used without engaging the ultrasound. The nozzle air temperatures (40, 50, and 60 °C) and airspeed (3 m/s) were the same for both SJR and SJR + US drying.

*Hot air drying:* Hot air drying was performed at the same air temperatures (40, 50, and 60 ℃) using the SJR + US device without turning on the ultrasound and SJR. Instead, a uniform airflow (3 m/s) was directed horizontally over the sample surfaces by a fan to remove moisture.

*Freeze drying:* The sweet potato samples were first frozen at –40 ℃ for over 24 h. Subsequently, the frozen samples were transferred to a freeze dryer (Harvest Right Inc., Utah, USA) with a vacuum pump set to around 0.08 mBar pressure, where they underwent freeze drying for 48 h.

### 2.3 Rehydration and shrinkage ratios

Dried sweet potato samples (1 g) were soaked in 100 mL of distilled water at 25 °C for 60 min. After removing excess water with absorbent paper, the rehydration ratio was calculated by reweighing the sweet potato pieces [24].

(1) $$Rehdrationratio\left(\%\right)=\frac{R_{2}}{R_{1}}\times 100$$

where R_1_ and R_2_ are the weight of dried sample (g) and weight of rehydrated sample (g), respectively.

The shrinkage of dried sweet potatoes over time was assessed using the liquid displacement method with toluene. Average values were obtained from three measurements, and shrinkage was calculated using the following formula.

(2) $$Shrinkageratio\left(\%\right)=\frac{V_{0}-V}{V_{0}}\times 100$$

where V_0_ denotes the volume before drying and V represents the volume after drying of the sweet potatoes.

### 2.4 Nutrient analysis

#### 2.4.1 Total starch content

Total starch content was assessed using AOAC method 996.11 (AOAC, 2007). Ground sweet potato samples (100 mg) were mixed with 0.2 mL of 80 % ethanol and 3.0 mL of thermostable α-amylase, then boiled for 6 min. After a 5-minute incubation at 50 °C, 0.1 mL of amyloglucosidase was added and incubated at room temperature for 30 min. The mixture was diluted to 10 mL with water, centrifuged (MegaStar 600R, VWR, Leuven, Belgium) at 1800×*g* for 10 min at 25 °C, and a 1.0 mL supernatant aliquot was further diluted to 10 mL. From this, 0.1 mL was mixed with 3 mL of glucose oxidase/peroxidase reagent and incubated at 50 °C for 20 min. Absorbance at 510 nm was measured using an Epoch microplate spectrophotometer (BioTek Instruments, Inc.).

#### 2.4.2 Soluble, insoluble, and total dietary fiber

An enzymatic–gravimetric method, following American Association of Cereal Chemists [1] guidelines, was used to measure soluble and insoluble dietary fiber in dried sweet potato samples. One gram of sample was treated with 100 mL of heat-resistant alpha-amylase (2.5 mg/mL), protease (2 mg/mL), and amyloglucosidase (0.5 mg/mL) at 95 °C for 30 min to break down starches and proteins, leaving the fiber intact. Insoluble dietary fiber (IDF) was collected after filtration, dried at 60 °C for 24 h, and the weight recorded. Soluble dietary fiber (SDF) was precipitated with 95 % ethanol (4 mL ethanol per 1 mL of filtrate), treated with ethanol and acetone, filtered, and collected. Both fiber fractions were dried at 60 °C for 24 h, weighed, and their weights summed to determine the total dietary fiber (TDF) content.

#### 2.4.3 β-Carotene

The β-carotene level in ground dried sweet potato samples was determined using the method by Yildiz [67]. One gram of dried sweet potato was mixed with 10 mL of hexane (6:4, v/v) until fully dispersed, and the original coloration disappeared. The mixture was then centrifuged at 6000 rpm for 10 min to separate the supernatant. The absorbance of the supernatant was measured at 453 nm, 505 nm, 645 nm, and 663 nm using a spectrophotometer. The β-carotene level was calculated using the following equation:

(3) $$\beta -Carotene\;\left(\frac{mg}{100g}\right)=\frac{A\times D\times F\times V\times MW\times 1000}{\in \times l\times W}$$

where A is the absorbance at the specified wavelength, D is the dilution factor, F is the correction factorYildiz (2022, V is the extract volume in mL, MW is the molecular weight of β-carotene (536.88 g/mol), ∊ is the molar absorptivity in hexane (2592 L/mol·cm), l is the cuvette path length, and W is the sample weight.

#### 2.4.4 Vitamin C (Ascorbic acid)

One gram of homogenized sweet potato slices was mixed with an extraction solution (2 g oxalic acid per 100 g sweet potato) and diluted to 100 mL. After a 10-minute extraction, the mixture was filtered. The supernatant was titrated with 2,6-dichlorophenol indophenol until a pink color appeared, marking the endpoint. The ascorbic acid content was calculated based on the volume of titrant used and its concentration, expressed as mg per 100 g of dry sample [24].

#### 2.4.5 Preparation of sample extracts

One gram of dried sweet potato was mixed with 4.5 mL of 80/20 methanol/water at 25 °C and agitated for 2 h in a water bath (SHEL LAB). After agitation, the mixture was centrifuged at 3000×*g* for 15 min using a MegaStar 600R centrifuge (VWR, Leuven, Belgium) to separate the supernatant. The supernatant was then filtered through Whatman filter paper No. 1 to clarify it for subsequent analysis. This sample preparation method follows the procedure outlined by Yildiz & Izli [64] for assessing the antioxidant capacity and total phenolic content of sweet potatoes.

#### 2.4.6 DPPH free radical scavenging activity

Sweet potato extracts’ antioxidant capacity was assessed using the DPPH radical scavenging assay. Sample extract (0.1 mL) was mixed with 25 mM DPPH methanolic solution (3.9 mL), incubated at 25 °C for 30 min, and absorbance was read at 517 nm. Antioxidant capacity was determined using a Trolox calibration curve (0.5 to 2.5 mM, y = 0.1267x – 0.0801, R2 = 0.9783), expressed as micromoles of Trolox equivalents per gram dry weight (d.w.) of sweet potato slices, following Yildiz & Izli's [64] method.

#### 2.4.7 Total phenolic content (TPC)

The total phenolic content (TPC) of dried sweet potato slices was determined using a method adapted from Yildiz [66] with gallic acid as a standard. Sweet potato extract (0.25 mL) was mixed with Folin–Ciocalteu reagent (1.25 mL) and 7.5 % Na_2_CO_3_ solution (3.75 mL), incubated for 2 h in darkness, and then measured at 765 nm. TPC was quantified using a gallic acid calibration curve (5 to 50 mg/L, y = 0.0707x – 0.1407, R^2^ = 0.9914), with results expressed as mg gallic acid equivalents per 100 g dry weight (d.w.) of sweet potato slices.

#### 2.4.8 Total flavonoid content (TFC)

The TFC of dried sweet potato samples was measured using a method based on the formation of a yellow complex with aluminum chloride, following Amagloh et al. [5]. Methanol extract (1 mL), diluted appropriately, was mixed with distilled water (4 mL), followed by sodium nitrite solution (3 mL, 15 g/100 mL), sodium hydroxide solution (4 mL, 4 g/100 mL), and methanolic aluminum chloride solution (0.3 mL, 10 g/100 mL). After adjusting the volume to 10 mL with distilled water, the solution stood for 15 min, and absorbance was measured at 510 nm. TFC was quantified using a quercetin standard curve, expressing results as mg quercetin equivalents (QE) per gram dry weight of dried sweet potato samples.

### 2.5 Color Measurement

Color changes in dried sweet potato slices were analyzed using an UltraScan VIS Spectrophotometer (HunterLab, Reston, VA, USA) equipped with EasyMatch QC software. Measurements in L*, a*, and b* color space (representing lightness/darkness, redness/greenness, and yellowness/blueness) were conducted after calibration with a black and white tile. Samples were measured three times at room temperature following Yildiz's [67] method. Total color difference (ΔE) values were calculated to quantify changes.

(4) $$\Delta E=\sqrt{{\left(L^{*}-L_{0}^{*}\right)}^{2}+{\left(a^{*}-a_{0}^{*}\right)}^{2}+{\left(b^{*}-b_{0}^{*}\right)}^{2}}$$

### 2.6 Glass transition temperature (Tg) and specific heat change (ΔCp)

Dried sweet potato samples were ground into powder (approximately 4 ± 1 mg) and placed in hermetically sealed aluminum pans for differential scanning calorimetry (DSC). The samples were cooled to −50 °C at 10 °C/min, held for 1 min, and then heated from −50 °C to 80 °C at 10 °C/min to analyze their thermal behavior. An empty aluminum pan served as the reference, with nitrogen gas (50 mL/min) used as the purge gas. This study, following Goula et al. [20], aimed to determine the glass transition temperature (Tg) and changes in specific heat capacity (ΔCp) of dried sweet potatoes.

### 2.7 Textural attributes

The textural characteristics of dried sweet potatoes were analyzed using a TA. XT Plus Texture Analyzer (Stable Micro Systems, Surrey, UK). A cylindrical probe with a 5 mm tip diameter was used to test the sweet potato pieces under fixed test parameters: a pre-speed of 2 mm/s, post-speed, and test speed of 0.5 mm/s, and a trigger force of 0.098 N. Two parameters, hardness and the number of peaks, were quantified and recorded. Each experiment was conducted three times, and the averaged outcomes were reported [64].

### 2.8 Fourier transform infrared spectra (FTIR)

The FTIR spectra of all dried sweet potatoes were obtained using a FTIR Spectrometer (Cary 670 FTIR, Agilent Technologies, Santa Clara, CA, USA) with a microscope attachment (Cary 610 FTIR, Agilent Technologies). The analysis involved dried sweet potato samples (0.1 g each), which were scanned 16 times within a spectral range of 4000–400 cm^−1^. The scans were conducted at a speed of 25 kHz, with a resolution of 4 cm^−1^ and a sensitivity setting of 1. Data acquisition and processing were performed utilizing Agilent Resolutions Pro software [49].

### 2.9 Data analysis and multivariate techniques

Each treatment was replicated three times unless stated otherwise. Statistical analyses were performed using JMP software (Version 7.0, SAS Institute Inc., Cary, NC, USA), employing Fisher’s LSD test at α = 0.05 to assess mean differences. In addition to the statistical analyses, principal component analysis (PCA) and heatmap analysis were conducted to evaluate the nutritional properties of dried samples. Principal component analysis (PCA) and heatmap generation were performed using Google Colab, an online Python-based environment built on Jupyter Notebook. This platform facilitated the execution of Python code for data manipulation and visualization. The PCA was conducted using the sklearn library, while heatmaps were created using the seaborn and matplotlib libraries to visually represent the nutritional composition data.

## 3 Discussion and results

### 3.1 Drying time reduction

Table 1 presents the drying times and drying time reductions of SJR + US, SJR, and HAD at three temperatures. Drying times were significantly reduced when ultrasound and/or air impinging by SJR was applied. Compared to HAD, SJR alone was effective in shortening the drying time, reducing it by 46 % at 40 °C and 33 % at both 50 °C and 60 °C, respectively (Table 1). The most significant reductions were observed for the combined SJR and US method. When ultrasound was applied, SJR + US recorded drying time reductions of 68 % at 40 °C, 58 % at 50 °C, and 50 % at 60 °C. In this experiment, both HAD and SJR involve using hot air to remove moisture and supply the energy needed for coupled heat and mass transport in sweet potato drying. The differences between HAD and SJR lie in the fact that in a HAD process, air is supplied to flow over the top surface of sweet potato slices whereas in SJR, the air is allowed to impinge onto sample surfaces via a specially designed SJR nozzle. The SJR nozzle enhances heat and mass transfer by directing a jet flow outward from the nozzle, which then reattaches to product surface. This reattachment process creates strong turbulent mixing at the boundary layer, improving the efficiency of heat and mass transfer [8]. On the other side, the addition of high frequency vibration introduces a further enhancement of drying process, which has been reported in a few recent studies using contact drying method [28], [32]. The enhancement by ultrasound can be attributed to at least 2 proposed mechanisms. When the moisture content in the sample is high, free water can be removed in the form of ultrasound-generated mist from product surface due to capillary waves and/or cavitation activities [59]. The biopolymeric matrices in sweet potato, acting as a non-saturated porous medium, undergo periodic vibrations that push the liquid in the pores to the surface, known as the sponge effect [38]. The combined action of these 2 mechanisms contributes to an enhanced moisture removal in direct contact-type ultrasonic drying.

**Table 1:** Drying time and efficiency of SJR + US, SJR, and HAD at different temperatures.

**Samples**	**Temperature (^o^C)**	**Drying time (min)**	**Drying time reduction (%)**
SJR + US	40	105	68
SJR	40	180	46
HAD	40	330	−
SJR + US	50	75	58
SJR	50	120	33
HAD	50	180	−
SJR + US	60	45	50
SJR	60	60	33
HAD	60	90	−

### 3.2 Rehydration and shrinkage ratios of dried sweet potato samples

**Fig. 2:**
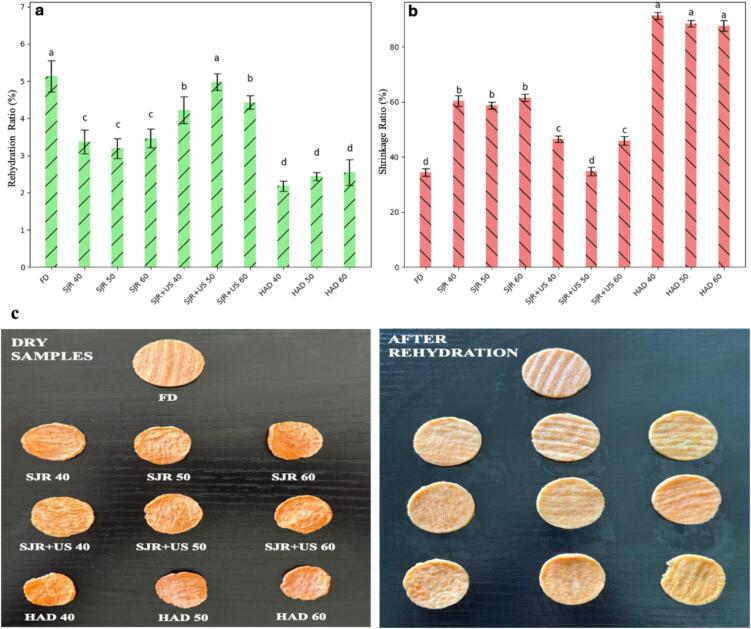
A) rehydration ratios of dried sweet potato samples, b) shrinkage ratios of dried sweet potato samples, c) effect of drying methods and rehydration on physical appearance of samples. *Note:* FD refers to freeze-dried sweet potatoes; SJR 40, SJR 50, and SJR 60 refer to sweet potatoes dried with slot jet reattachment (SJR) nozzle at 40 °C, 50 °C, and 60 °C, respectively; SJR + US 40, SJR + US 50, and SJR + US 60 represent sweet potatoes dried with ultrasound (US) and slot jet reattachment (SJR) nozzle at 40 °C, 50 °C, and 60 °C, respectively; HAD 40, HAD 50, and HAD 60 correspond to sweet potatoes dried with hot air drying (HAD) at 40 °C, 50 °C, and 60 °C, respectively.

Rehydration ratios (RR) and shrinkage are two comprehensive quality parameters for evaluating the overall quality of dried food products. The RR values of dried sweet potato samples are shown in Fig. 2a. A good rehydration ability, as indicated by a high RR value, suggests less irreversible cellular structure damage. This allows sweet potato cells to effectively regain water lost during drying, reflecting a better product quality. The sweet potato samples dried using US + SJR showed significantly higher RR (P ≤ 0.05) compared to those dried by hot-air drying (HAD). Specifically, SJR + US at 50 °C achieved a RR of 4.98, which is very close to that of the freeze-dried samples (5.13). The sponge effect associated with ultrasonic drying may promote the formation of microchannel within the polymeric matrix [31], which may result in increased porosity and cell membrane permeability in dried sweet potatoes. This contributes to the rapid and better rehydration of SJR + US samples, outperforming the HAD samples (Fig. 2a). These findings are consistent with the results of Ignaczak et al. [23], which showed that hot-air drying of carrots reduced RR due to shrinkage and reduced porosity, both of which hinder rehydration. Similarly, studies by Szadzińska et al. [55], Zhao et al. [69], and Tao et al. [56] reported that ultrasound treatment improves the rehydration capability of dried foods, helping restore texture and overall quality.

The shrinkage ratio (SR) of sweet potatoes dried by four methods follows a similar pattern to their rehydration ratios (Fig. 2b). Sweet potatoes exposed to HAD showed significantly higher shrinkage rates (≥87.6 %) compared to other methods (Fig. 2b). The highest SR occurred in HAD samples at 40 °C (91.3 %). As the temperature increased from 40 °C to 60 °C, a slight but non-significant decline in SR (P > 0.05) was observed. HAD is a lengthy process, and the samples were exposed to high temperatures for an extended period. As a result, the products experienced cellular shrinkage and tissue damage, consequently reducing water diffusion during rehydration [46]. Shrinkage in food products is generally undesirable because it alters appearance and shape, reduces volume, and increases firmness, all of which affect consumer perception. In contrast, freeze-drying (FD) offers advantages for water removal due to its low-temperature processing, which better preserves product quality, including hydration, flavor, and shape [10]. As seen in Fig. 2b, freeze-dried sweet potatoes exhibited the lowest shrinkage rate (34.4 %), followed by SJR + US drying (34.8–46.5 %) and SJR alone (58.7–61.5 %). SJR + US at 50 °C resulted in a shrinkage ratio similar to freeze-drying (34.8 %), with no significant difference between the two methods (P > 0.05). Therefore, ultrasonic drying is effective in preserving the physical quality attributes, as evidenced by high rehydration and low shrinkage.

### 3.3 Nutrient composition

The study highlighted significant variations in total phenolic content (TPC) of dried sweet potato slices depending on the drying technique and temperature. SJR + US at 50 °C achieved the highest TPC (158.19 mg GAE/100 g), closely followed by freeze-drying at 157.65 mg GAE/100 g, with no significant difference between them (Table 2). The lowest TPC (126.45 mg GAE/100 g) was observed in HAD samples at 40 °C. Higher temperatures and prolonged exposure to heat, especially in hot-air drying, typically result in the degradation of naturally occurring phenolic compounds in foods, unless thermal transformation or the formation of Maillard reaction-derived phenolics occurs [16]. This likely explains the low TPC observed in all HAD samples (Table 2). In US-treated samples (SJR + US), cavitation-driven activities may help liberate polyphenols trapped within cell walls and deactivate oxidative enzymes, leading to significantly higher TPC values compared to other treatments. In comparison, freeze-drying preserves TPC by operating at low temperatures and in oxygen-free conditions. The formation of ice crystals during freeze-drying disrupts cell structures, aiding phenolic release [3]. Therefore, selecting appropriate drying methods and processing conditions is crucial for retaining bioactive compounds in sweet potatoes, enhancing their nutritional and functional value.

**Table 2:** Nutrient composition of dried sweet potato samples.

**Samples**	**TPC** **(mg GAE/100** **g)**	**TFC** **(QE mg/g d.w)**	**DPPH** **(μmol TE/g)**	**TFC** **(QE mg/g d.w)**	**β-Carotene (mg/100 g)**	**Vitamin C (mg/100 g)**
**FD**	157.65 ± 0.51^a^	118.44 ± 0.23^a^	6.02 ± 0.23^a^	118.44 ± 0.23^a^	35.12 ± 0.24^a^	5.44 ± 0.05^a^
**SJR 40**	138.43 ± 0.61^c^	93.19 ± 0.42^b^	4.67 ± 0.09^c^	93.19 ± 0.42^b^	25.15 ± 0.48^c^	4.42 ± 0.13^c^
**SJR 50**	134.22 ± 0.13^d^	96.25 ± 0.55^b^	4.18 ± 0.33^c^	96.25 ± 0.55^b^	26.03 ± 0.33^c^	4.35 ± 0.07^c^
**SJR 60**	139.48 ± 0.89^c^	95.54 ± 0.71^b^	5.55 ± 0.21^b^	95.54 ± 0.71^b^	30.34 ± 0.74^b^	4.46 ± 0.04^c^
**SJR + US 40**	154.34 ± 0.48^b^	113.51 ± 0.77^a^	6.05 ± 0.07^a^	113.51 ± 0.77^a^	30.83 ± 0.65^b^	4.88 ± 0.05^b^
**SJR + US 50**	158.19 ± 0.75^a^	119.08 ± 0.09^a^	6.44 ± 0.11^a^	119.08 ± 0.09^a^	31.98 ± 0.13^b^	5.27 ± 0.11^a^
**SJR + US 60**	155.79 ± 0.16^b^	115.15 ± 0.13^a^	6.09 ± 0.15^a^	115.15 ± 0.13^a^	30.07 ± 0.09^b^	4.91 ± 0.22^b^
**HAD 40**	126.45 ± 0.33^f^	39.37 ± 0.38^c^	2.22 ± 0.12^e^	39.37 ± 0.38^c^	13.43 ± 0.22^e^	3.02 ± 0.08^e^
**HAD 50**	127.13 ± 0.08^f^	39.41 ± 0.86^c^	3.09 ± 0.09^d^	39.41 ± 0.86^c^	14.27 ± 0.27^e^	3.85 ± 0.01^d^
**HAD 60**	129.68 ± 0.41^e^	42.99 ± 0.12^c^	3.14 ± 0.02^d^	42.99 ± 0.12^c^	18.76 ± 0.48^d^	3.93 ± 0.02^d^

TPC: Total phenolic content; TFC: Total flavonoid content.

^a-f^ Values are means ± standard deviation (*n* = 3), and values followed by the same letters in the same column are not significantly different (P ≤ 0.05).

The total flavonoid content (TFC) of dried sweet potatoes varied significantly across different drying techniques (Table 2). The SJR + US samples consistently exhibited the highest TFC values when dried at 50 °C (119.08 QE mg/g d.w). In contrast, the lowest TFC values were found in HAD sweet potatoes at 40 °C (39.37 QE mg/g d.w). These findings highlight the significant impact of drying techniques and temperatures on flavonoid preservation in sweet potatoes. No significant differences were observed between freeze-dried and SJR + US dried sweet potatoes, underscoring the effectiveness of SJR + US in retaining flavonoid content compared to SJR alone and HAD methods. Previous studies have also demonstrated the positive role of ultrasound (US) in preserving flavonoid content in various food products, including sweet potatoes [48]. In contrast, studies on other fruits and vegetables such as papaya, tomato, and Chinese ginger have shown that high-temperature drying methods like HAD can lead to a significant decline in flavonoid levels due to oxidative decomposition during prolonged drying times [6]. The oxidative decomposition of flavonoids during HAD processes highlights the importance of minimizing oxidation pathways to retain bioactive compounds in dried sweet potatoes. The decline in flavonoid content not only impacts the nutritional quality but also diminishes the potential biological activities of dried sweet potato products.

Similar to TPC and TFC, the sweet potato dried with SJR + US at 50 °C exhibited the highest DPPH activity (10.44 μmol TE/g), while those dried using hot air at 40 °C had the lowest (1.88 μmol TE/g) (Table 2). The low DPPH activity in HAD samples may result from reduced retention of phenolic compounds. Increasing drying temperatures from 40 to 60 °C increased DPPH activity, likely due to the release of bound polyphenols, as noted by Antony & Farid [7]. Samples dried with the SJR nozzle had lower DPPH activity than the SJR US and freeze-dried samples but higher than the HAD samples. SJR + US drying showed no significant differences in antioxidant activity compared to freeze-dried samples (P > 0.05), indicating good retention of phenolic compounds. The highest antioxidant activity (SJR + US at 50 °C) correlated with the highest total phenolic content (TPC) in Table 2, suggesting that DPPH radical scavenging is influenced by polyphenols' hydrogen donor properties. Numerous studies, including those on quince, pomelo, and red pepper, have documented a positive relationship between total phenolic concentration and antioxidant activity, highlighting the important role of phenolic compounds in enhancing antioxidative properties [64], [65], [12]. Yildiz [66] noted that the enhanced antioxidant capacity in dried fruits and vegetables often arises from synergistic interactions among natural phenolic compounds.

The vitamin C levels in sweet potato samples were significantly reduced by HAD (Table 2). While sweet potatoes provide about 20–30 % of the recommended daily allowance of vitamin C per serving, making them a decent source [4], heat treatment markedly decreases these levels. The lowest vitamin C content was found in HAD samples at 40 °C, with all HAD samples showing significantly lower vitamin C levels compared to other drying methods. The oxidative and hydrolytic reactions during HAD contribute to the degradation and reduction of vitamin C in dried products [18]. Freeze-dried sweet potato samples had the highest vitamin C level (5.44 mg/100 g), followed closely by SJR + US drying at 50 °C (5.27 mg/100 g), with no significant difference between these two methods. Vitamin C is prone to oxidation, especially when food is exposed to air during drying [68]. Ultrasonic drying may reduce oxidation by speeding up moisture removal, thereby decreasing exposure time to oxygen [22]. Additionally, ultrasonic drying with MMM variable technology (see section 2.2) promotes uniform drying throughout the sweet potato, which helps maintain consistent vitamin C levels and minimizes localized degradation [32].

The β-Carotene levels in dried sweet potato samples ranged from 8.3 to 156.6 mg/100 g (Table 2). Freeze-dried samples retained the highest β-Carotene (35.12 mg/100 g), followed by SJR + US drying at 50 °C (31.98 mg/100 g). HAD reduced β-Carotene levels due to heat exposure and prolonged drying timeGonçalves et al. [19]. Although research on ultrasound drying’s effect on β-Carotene in sweet potatoes is limited, its mild processing conditions and short drying time are likely to preserve cellular structure, thereby protecting β-Carotene [50]. Data in Table 2 indicate that β-Carotene levels were significantly higher in SJR + US samples compared to those treated with HAD or SJR nozzle (P ≤ 0.05). HAD-treated samples exhibited the lowest β-Carotene levels due to degradation through heat-induced molecular isomerization, shifting carotenoids from their all-trans to cis forms at specific carbon positions [67]. This degradation is compounded by oxidative reactions caused by increased surface exposure during drying (Ruttarattanamongkol, 2016). β-Carotene levels correlate with L* (lightness) and b* (yellowness) color values, enhancing the sweet potatoes' yellow-orange color [40]. Samples with higher β-Carotene, such as those dried by SJR + US at 50 °C, also had higher L* (75.29) and b* values. Conversely, HAD samples at 40 °C had the lowest β-Carotene (13.43 mg/100 g) and correspondingly lower L* (68.73) and b* (20.42) values.

Table 3 tabulates the preservation of total starch content across different drying methods and temperatures. SJR + US at 50 °C achieved the highest starch content (36.84 g/100 g), likely due to reduced heat damage and enhanced starch integrity. SJR + US also performed well at 40 °C (35.77 g/100 g) and 60 °C (36.03 g/100 g), close to that of freeze-drying (36.57 g/100 g). SJR alone showed variable results, ranging from 31.11 to 33.43 g/100 g across temperatures, indicating moderate heat sensitivity. In contrast, HAD retained the least starch, with values from 26.96 to 28.65 g/100 g (Table 3). The drying process, especially if done at high temperatures and long time, can lead to the breakdown of starch into simple sugars [60]. This is due to the activation of enzymes like amylases that break down starches as part of the drying procedure [44]. US drying is known for its potential to reduce drying time and enhance overall drying effectiveness. The rapid drying process can minimize the exposure of starch to prolonged heat and oxygen, which could help retain more of the original starch content in contrast to traditional drying techniques (Calín-Sánchez, 2020).

**Table 3:** Nutrient composition of dried sweet potato samples.

**Samples**	**Total Starch (g/100 g)**	**TDF** **(g/100 g)**	**IDF** **(g/100 g)**	**SDF** **(g/100 g)**
**FD**	36.57 ± 0.12^a^	8.57 ± 0.34^a^	5.84 ± 0.12^a^	2.73 ± 0.22^a^
**SJR 40**	33.15 ± 0.09^b^	5.42 ± 0.56^d^	3.33 ± 0.45^d^	2.09 ± 0.11^b^
**SJR 50**	31.11 ± 0.24^c^	5.30 ± 0.33^d^	3.26 ± 0.19^d^	2.04 ± 0.14^b^
**SJR 60**	33.43 ± 0.43^b^	5.23 ± 0.61^d^	3.21 ± 0.23^d^	2.02 ± 0.38^b^
**SJR + US 40**	35.77 ± 0.58^a^	6.74 ± 0.54^c^	4.03 ± 0.16^c^	2.71 ± 0.38^a^
**SJR + US 50**	36.84 ± 0.71^a^	8.48 ± 0.59^a^	5.69 ± 0.40^a^	2.79 ± 0.19^a^
**SJR + US 60**	36.03 ± 0.38^a^	7.55 ± 0.17^b^	4.82 ± 0.06^b^	2.73 ± 0.11^a^
**HAD 40**	26.96 ± 0.64^e^	2.65 ± 0.49^f^	1.73 ± 0.31^f^	0.92 ± 0.18^d^
**HAD 50**	28.33 ± 0.46^d^	3.82 ± 0.83^e^	2.34 ± 0.56^e^	1.48 ± 0.27^c^
**HAD 60**	28.65 ± 0.69^d^	3.89 ± 0.43^e^	2.40 ± 0.31^e^	1.49 ± 0.12^c^

TDF: Total dietary fiber; IDF: Insoluble dietary fiber; SDF: Soluble dietary fiber.

^a-f^ Values represent means ± standard deviation (n = 3), and values sharing the same letters within the same column are not significantly different (P ≤ 0.05).

Fiber intake is essential for health, comprising soluble and insoluble forms that contribute to the total dietary fiber (TDF) of foods. Soluble fiber, such as pectin, inulin, β-glucans, and gums, forms a gel-like substance in the digestive tract, stabilizing blood sugar levels, reducing cholesterol, and promoting heart health [21], [34]. Insoluble fiber, including lignin, cellulose, and certain hemicelluloses, enhances stool bulk, promoting regular bowel movements and preventing constipation (Maposa & Jideani, 2015). Sweet potatoes are rich in dietary fiber, with a daily recommended intake of 20 to 38 g according to World Health Organization (WHO) and the United States Food and Drug Administration (USFDA) [33]. Foods containing over 6 g of total dietary fiber per 100 g are classified as high in dietary fiberCAC [11]., and sweet potatoes meet this criterion (Table 3). An ideal balance of dietary fiber is often described as a 2:1 ratio of insoluble to soluble fiber, enhancing health benefits [36]. As shown in Table 3, freeze-drying best preserved TDF in sweet potatoes at 8.57 g/100 g due to low-temperature moisture removal. SJR + US treatment also improved TDF preservation (6.74–8.48 g/100 g) by maintaining cell wall integrity and limiting thermal degradation [53]. In contrast, hot air drying (HAD) resulted in the lowest TDF content (2.65–3.89 g/100 g) due to fiber degradation from high temperatures and long drying time (Table 1). Ultrasound demonstrated potential in enhancing fiber retention in dried sweet potatoes by reducing processing time, heat exposure, and oxidation.

**Fig. 3:**
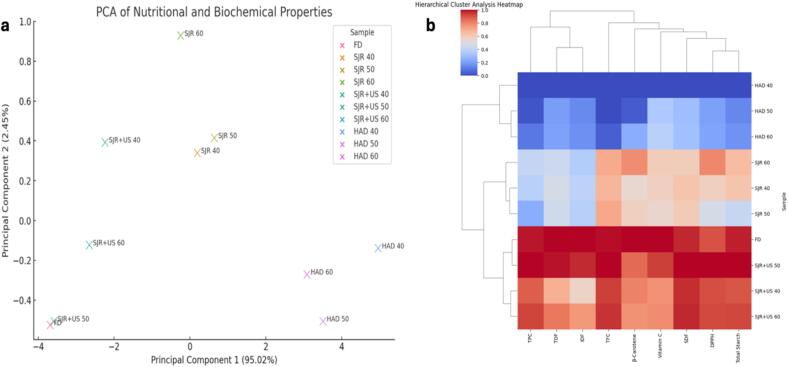
A)Principal component (PCA) analysis of Nutritional Properties, b) Nutritional Profiling of Various Drying Methods: Heatmap Analysis of TPC, DPPH, TFC, β-Carotene, Vitamin C, Starch, and Dietary Fiber Content.

In this study, principal component analysis (PCA) and heatmap visualization were used to compare and visualize the nutritional profiles of sweet potato samples subjected to different drying methods. PCA reduces high-dimensional data, such as nutritional profiles, into principal components that capture the majority of variability, helping identify key variables that influence differences between samples [27]. Complementing PCA, heatmaps provide a graphical representation of data, with color intensity indicating the magnitude of specific parameters, making it easy to identify clusters and trends [61]. Together, these techniques offer valuable insights into complex datasets, aiding in product development, quality assurance, and nutritional studies. As can be seen from the PCA profile (Fig. 3a), the PC1 and PC2 scores for each sample group were 95.02 % and 2.45 %, respectively. It is worth noting that there is a significant difference between HAD sample groups (especially HAD 40) and FD samples. On the other hand, SJR + US samples (especially SJR + US 50) closely grouped or overlapping with FD samples indicate similarities in their nutritional properties.

In addition, a heatmap was generated to visualize the comparative nutritional profiles of sweet potato samples subjected to various drying methods (Fig. 3b). The heatmap depicts key nutritional parameters including TPC, DPPH, TFC, β-Carotene, Vitamin C, Total Starch, and Dietary Fiber (TDF, IDF, SDF). Each row represents a different drying method, while columns correspond to the measured nutritional components. Color intensity in each cell reflects the concentration of the respective nutrient, with darker shades indicating higher values. As can be seen from the heatmap, treatments such as FD and SJR + US often show higher values (high color intensity) across multiple nutrients compared to others like HAD, suggesting these methods may better preserve or enhance nutritional content of sweet potatoes. These findings can guide decisions in food processing and product development, helping to optimize methods and conditions that maximize nutritional quality or provide to specific nutritional requirements.

### 3.4 Impact of drying techniques on color characteristics of sweet potatoes

Color is a crucial quality parameter for dried fruits, significantly impacting consumer acceptance [57]. Table 4 shows the color characteristics of dried sweet potatoes from various drying methods. Ultrasound contact drying, when combined with SJR, enhanced lightness (L* value), achieving the highest L* at 50 °C for SJR + US. Freeze-drying retained the highest overall L*, while HAD resulted in darker samples due to prolonged heat exposure [43]. Freeze-dried samples exhibited the lowest redness and highest yellowness, while HAD samples showed the opposite. Yellowness (b*) correlates with β-carotene content [52], with higher values in SJR + US dried samples. The highest b* values and β-carotene content were observed in freeze-dried and SJR + US samples, while HAD samples had the lowest (Table 4 and Table 2). Wang et al. [58] observed a comparable effect in ultrasound-treated carrot cells, where b* values increased significantly compared to untreated samples. ΔE values indicated total color changes, with SJR + US samples showing the smallest ΔE, followed by SJR, and HAD exhibiting the highest ΔE. Djekic et al. [14] and Karaman et al. (2021) remarked that HAD demonstrated higher ΔE values in comparison to FD samples. Color changes resulted from factors like the Maillard reaction and pigment breakdown [64], [65]. Shorter drying times in SJR and SJR + US enhanced color retention compared to HAD. Ultimately, the non-thermal nature and efficient mass transfer of ultrasound drying better preserved color by minimizing heat-sensitive pigment degradation [51].

**Table 4:** The color attributes of dried sweet potato samples with different drying methods.

**Samples**	L*	a*	b*	***ΔE***
FD	71.64 ± 0.13^a^	18.24 ± 0.22^d^	23.74 ± 0.19^a^	−
SJR 40	61.34 ± 0.84^d^	20.07 ± 0.38^b^	21.02 ± 0.03^c^	10.73 ± 0.86^d^
SJR 50	61.07 ± 0.11^d^	20.42 ± 0.12^b^	21.37 ± 0.46^c^	11.05 ± 0.19^c^
SJR 60	61.85 ± 0.09^d^	20.03 ± 0.29^b^	21.05 ± 0.94^c^	10.31 ± 0.17^d^
SJR + US 40	64.64 ± 0.86^c^	19.55 ± 0.14^c^	22.33 ± 0.19^b^	7.26 ± 0.03^e^
SJR + US 50	68.29 ± 0.73^b^	18.37 ± 0.65^d^	23.42 ± 0.25^a^	3.37 ± 0.29^f^
SJR + US 60	64.78 ± 0.45^c^	19.49 ± 0.72^c^	22.56 ± 0.14^b^	7.07 ± 0.34^e^
HAD 40	58.01 ± 0.49^e^	21.44 ± 0.19^a^	20.19 ± 0.44^d^	14.44 ± 0.78^a^
HAD 50	58.35 ± 0.56^e^	20.32 ± 0.74^b^	21.15 ± 0.21^c^	13.69 ± 0.33^b^
HAD 60	58.81 ± 0.31^e^	20.31 ± 0.08^b^	21.22 ± 0.36^c^	13.23 ± 0.76^b^

### 3.5 Glass transition temperature (Tg) and specific heat change (ΔCp)

Table 5 outlines the glass transition temperatures (Tg) of sweet potatoes dried by 4 methods (FD, SJR, SJR + US, and HAD). Tg is the change from a hard, glass-like state to a soft, rubber-like state, occurring within a 10 to 20 °C range for amorphous sugars [39], [47]. Higher Tg values suggest improved storage stability, with FD and SJR + US samples exhibiting significantly higher Tg compared to SJR and HAD samples, indicating better room temperature stability. The lower Tg in HAD samples is attributed to longer drying times and higher temperatures, leading to structural damage [26]. Table 5 also presents specific heat capacity change (ΔCp) during glass transition, showing that lower Tg values correspond to lower ΔCp values. SJR and SJR + US samples had higher ΔCp values (1.06–1.16 J/g °C) compared to HAD samples (0.95–0.96 J/g °C), suggesting a greater proportion of non-crystalline solids [37]. Similar trends were reported by Kahraman et al. [28] in apple slices subjected to freeze-drying, hot air drying, and non-thermal ultrasound drying methods.

**Table 5:** Glass transition temperature (Tg), specific heat capacity change (ΔCp), and texture characteristics of sweet potatoes dried using different methods.

**Samples**	**Thermal stability**		**Texture**	
	**Tg (^o^C)**	**ΔCp (J/g ^o^C)**	**Hardness (*N*)**	**Number of Peaks**
**FD**	15.22 ± 0.76^a^	1.29 ± 0.19^a^	11.07 ± 0.05^c^	19.20 ± 0.04^a^
**SJR 40**	13.54 ± 0.33^ab^	1.06 ± 0.01^c^	16.41 ± 0.11^b^	5.40 ± 0.39^b^
**SJR 50**	12.91 ± 0.12^ab^	1.04 ± 0.07^c^	16.55 ± 0.07^b^	5.10 ± 0.43^b^
**SJR 60**	13.18 ± 0.09^ab^	1.05 ± 0.08^c^	15.93 ± 0.13^b^	6.70 ± 0.11^b^
**SJR + US 40**	14.09 ± 0.13^a^	1.16 ± 0.05^b^	15.96 ± 0.22^b^	6.80 ± 0.19^b^
**SJR + US 50**	14.49 ± 0.05^a^	1.19 ± 0.17^b^	15.13 ± 0.02^b^	6.10 ± 0.15^b^
**SJR + US 60**	14.16 ± 0.09^a^	1.18 ± 0.22^b^	15.48 ± 0.01^b^	6.40 ± 0.22^b^
**HAD 40**	10.43 ± 0.16^b^	0.95 ± 0.46^d^	18.99 ± 0.06^a^	1.30 ± 0.03^c^
**HAD 50**	10.65 ± 0.53^b^	0.93 ± 0.38^d^	18.43 ± 0.05^a^	1.70 ± 0.01^c^
**HAD 60**	10.31 ± 0.12^b^	0.96 ± 0.63^d^	18.34 ± 0.17^a^	1.80 ± 0.07^c^

^a-d^ Values are presented as means ± standard deviation (n = 3), and values sharing the same letters within the same column are not significantly different (P ≤ 0.05).

### 3.6 Texture

Table 5 presents two key texture parameters—hardness and number of peaks—for sweet potatoes dried using FD, SJR, SJR + US, and HAD. Texture is crucial for consumer satisfaction, with higher hardness indicating a firmer product [54]. In this study, the drying method significantly influenced hardness (P ≤ 0.05), with freeze-dried sweet potatoes showing the lowest hardness (11.07 N) compared to SJR, SJR + US, and HAD. Freeze-drying resulted in increased porosity, affecting rehydration capacity and texture (Mounir et al., 2015), leading to a softer, more brittle structure due to numerous pores. Sweet potatoes dried with SJR + US had hardness values closer to freeze-dried samples. In contrast, HAD samples exhibited the highest hardness (18.99 N, 18.43 N, and 18.34 N at 40, 50, and 60 °C, respectively), consistent with findings from Kahraman et al. [28]. Crispness, indicated by the total number of peaks [15] was significantly higher in freeze-dried, SJR, and SJR + US samples compared to HAD. The migration of surface moisture in HAD contributes to a firmer texture and a hard outer surface, a phenomenon known as case-hardening [45], which likely explains the increased hardness and number of peaks in these samples.

### 3.7 FTIR analysis

**Fig. 5:**
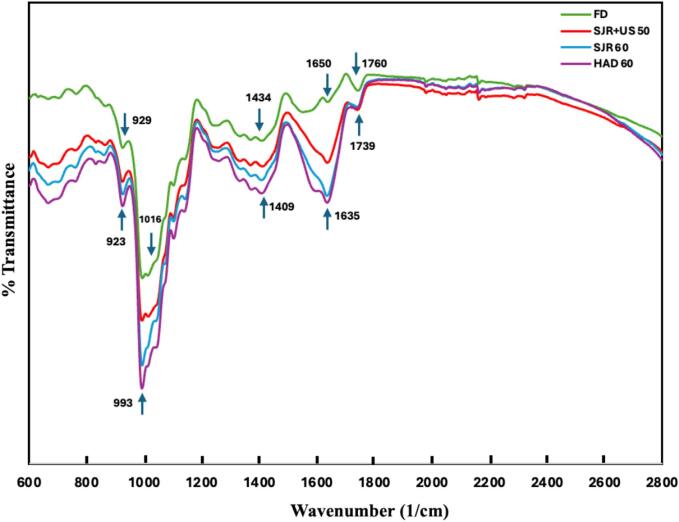
FTIR spectra of dried sweet potato samples.

FTIR spectroscopy provides valuable insights into the chemical composition, structural integrity, and process optimization of dried food products [17]. The FTIR analysis of sweet potato samples dried using four different methods (FD, HAD-60, SJR-60, and SJR + US-50) highlighted notable differences in their chemical structures and the preservation of bioactive compounds (Fig. 5). In the polysaccharide region (∼900–1200 cm^−1^), FD displayed the highest peak intensities at 929 and 1016 cm^−1^, indicating superior retention of polysaccharides due to its non-thermal drying mechanism. In comparison, the peaks for SJR + US-50 (927 and 1012 cm^−1^) exhibited slight shifts, suggesting mild structural alterations. Both SJR-60 and HAD-60 showed significant reductions and shifts in this region, indicative of polysaccharide degradation caused by thermal processing. The amide I region (∼1600–1700 cm^−1^), which represents proteins, showed the most prominent peak at 1650 cm^−1^ in FD, highlighting effective preservation of protein structure. SJR + US-50 showed a minor shift to 1646 cm^−1^, reflecting minimal protein degradation. Conversely, HAD-60 exhibited substantial shifts and reduced peak intensities at 1635 cm^−1^, signifying significant protein denaturation due to heat exposure during the drying process. In the lipid-associated region (∼1700–1800 cm^−1^), the C=O stretching peak was retained optimally in FD at 1760 cm^−1^. SJR + US-50 demonstrated a slight reduction and shift to 1747 cm^−1^, indicating partial lipid oxidation or structural modification. SJR-60 and HAD-60 (1739 cm^−1^) showed the lowest peak intensities in this region, suggesting considerable lipid degradation during drying. Overall, FD was the most effective method for preserving polysaccharides, proteins, and lipids, maintaining the structural integrity of the sweet potato samples across all key regions. SJR + US-50 also demonstrated good retention of bioactive compounds with minimal degradation, while HAD-60 and SJR-60 resulted in significant chemical degradation, making them less favorable options for preserving the nutritional quality and functionality of sweet potatoes.

## 4 Conclusion

This study explored the impact of a novel combination of slot jet reattachment (SJR) nozzle and ultrasound (US) drying (SJR + US) on the quality attributes of sweet potatoes, compared to freeze-drying (FD), SJR drying, and hot air drying (HAD) methods. SJR + US exhibited drying performance comparable to FD in terms of quality retention. Compared to HAD, SJR + US-dried sweet potatoes showed significant enhancements in physical properties (rehydration ratio and shrinkage) and nutritional composition (including total starch, TDF, TPC, TFC, DPPH, β-carotene, and vitamin C), as well as improvements in color and texture characteristics. Both FD and SJR + US methods resulted in significantly higher glass transition temperatures (Tg) of dried sweet potatoes compared to HAD, indicating their improved stability at room temperature. FTIR analysis, principal component analysis (PCA), and heatmap confirmed that SJR + US drying effectively preserved nutritional qualities, ensuring consistent product quality. In summary, SJR + US provides a promising alternative to traditional sweet potato drying methods, offering superior quality improvements in dried sweet potatoes.

## CRediT authorship contribution statement

**Gulcin Yildiz:** Writing – original draft, Methodology, Investigation, Formal analysis, Data curation. **Yuan Gao:** Investigation. **Junzhou Ding:** Investigation. **Si Zhu:** Investigation. **Guibing Chen:** Supervision, Resources. **Hao Feng:** Writing – review & editing, Supervision, Resources, Funding acquisition, Conceptualization.

## Declaration of competing interest

The authors declare that they have no known competing financial interests or personal relationships that could have appeared to influence the work reported in this paper.
